# Multicenter evaluation of gut microbiome profiling by next-generation sequencing reveals major biases in partial-length metabarcoding approach

**DOI:** 10.1038/s41598-023-46062-7

**Published:** 2023-12-18

**Authors:** Hugo Roume, Stanislas Mondot, Adrien Saliou, Sophie Le Fresne-Languille, Joël Doré

**Affiliations:** 1 Université Paris-Saclay, INRAE, MetaGenoPolis, 78350 Jouy-en-Josas, France; 2 Université Paris-Saclay, INRAE, AgroParisTech, Micalis Institute, 78350 Jouy-en-Josas, France; 3 Discovery & Front End Innovation, Lesaffre Institute of Science & Technology, Lesaffre International, 101 rue de Menin, 59700 Marcq-en-Barœul, France; 4 BIOASTER, Microbiology Technology Institute, 40 Avenue Tony Garnier, 69007 Lyon, France; 5 Biofortis SAS, 3 Route de la Chatterie, Saint-Herblain, 44800 Nantes, France

**Keywords:** Biotechnology, Microbiology, Molecular biology, Gastroenterology

## Abstract

Next-generation sequencing workflows, using either metabarcoding or metagenomic approaches, have massively contributed to expanding knowledge of the human gut microbiota, but methodological bias compromises reproducibility across studies. Where these biases have been quantified within several comparative analyses on their own, none have measured inter-laboratory reproducibility using similar DNA material. Here, we designed a multicenter study involving seven participating laboratories dedicated to partial- (P1 to P5), full-length (P6) metabarcoding, or metagenomic profiling (MGP) using DNA from a mock microbial community or extracted from 10 fecal samples collected at two time points from five donors. Fecal material was collected, and the DNA was extracted according to the IHMS protocols. The mock and isolated DNA were then provided to the participating laboratories for sequencing. Following sequencing analysis according to the laboratories’ routine pipelines, relative taxonomic-count tables defined at the genus level were provided and analyzed. Large variations in alpha-diversity between laboratories, uncorrelated with sequencing depth, were detected among the profiles. Half of the genera identified by P1 were unique to this partner and two-thirds of the genera identified by MGP were not detected by P3. Analysis of beta-diversity revealed lower inter-individual variance than inter-laboratory variances. The taxonomic profiles of P5 and P6 were more similar to those of MGP than those obtained by P1, P2, P3, and P4. Reanalysis of the raw sequences obtained by partial-length metabarcoding profiling, using a single bioinformatic pipeline, harmonized the description of the bacterial profiles, which were more similar to each other, except for P3, and closer to the profiles obtained by MGP. This study highlights the major impact of the bioinformatics pipeline, and primarily the database used for taxonomic annotation. Laboratories need to benchmark and optimize their bioinformatic pipelines using standards to monitor their effectiveness in accurately detecting taxa present in gut microbiota.

The development of next-generation high-throughput sequencing technologies has facilitated significant advances in microbial ecology, allowing the study of microbial communities at an unprecedented level of resolution, through the ability to profile their diversity and characterize their genetic information, without prior cultivation^1^. In addition to their significant impact on our understanding of life forms, DNA sequencing technologies, including metabarcoding and metagenomics, have opened up a new area of genomics for studying microbial ecosystems. Metabarcoding uses amplicon PCR sequencing, most often of the 16S rRNA gene as a phylogenetic marker that is restricted to bacteria and archaea. Metagenomics allows analysis of collective microbial genomes in their natural habitat, using shotgun sequencing, which captures the entire genetic information of a sample. While single-gene amplicon sequencing only allows exploration of the taxonomic diversity of prokaryotic taxa, sequencing the entire genomic content allows exploration of gene-encoded functions as well as information about the genomes of microorganisms from multiple prokaryotic taxa.

Both sequencing approaches have common biases and limitations, which have been reviewed^2^, such as those linked to sample collection^3^, biobanking^4^, contaminants^5^, the selection of DNA extraction protocols^6,7^, library preparation methods^8–10^ approach over metabarcoding in detecting low-abundance bacteria^36^.

None of these studies measured inter-laboratory reproducibility, using similar DNA material to measure biases generated by sequencing protocols and bioinformatics pipelines or sequencing protocols only. Here, we report an inter-laboratory reproducibility study of metabarcoding (P1 to P6) versus metagenomic profiling (MGP) approaches using similar DNA material extracted in triplicate following the Human Microbiome Standards (IHMS) protocol from 10 fecal samples collected from five donors at two time points as well as one mock DNA community sample. In order to assess specific biases due to sequencing protocols, raw sequences delivered by partners were reprocessed using a single bioinformatics pipeline.

## Methods

### Study design, participants, and sampling

**Figure 1:**
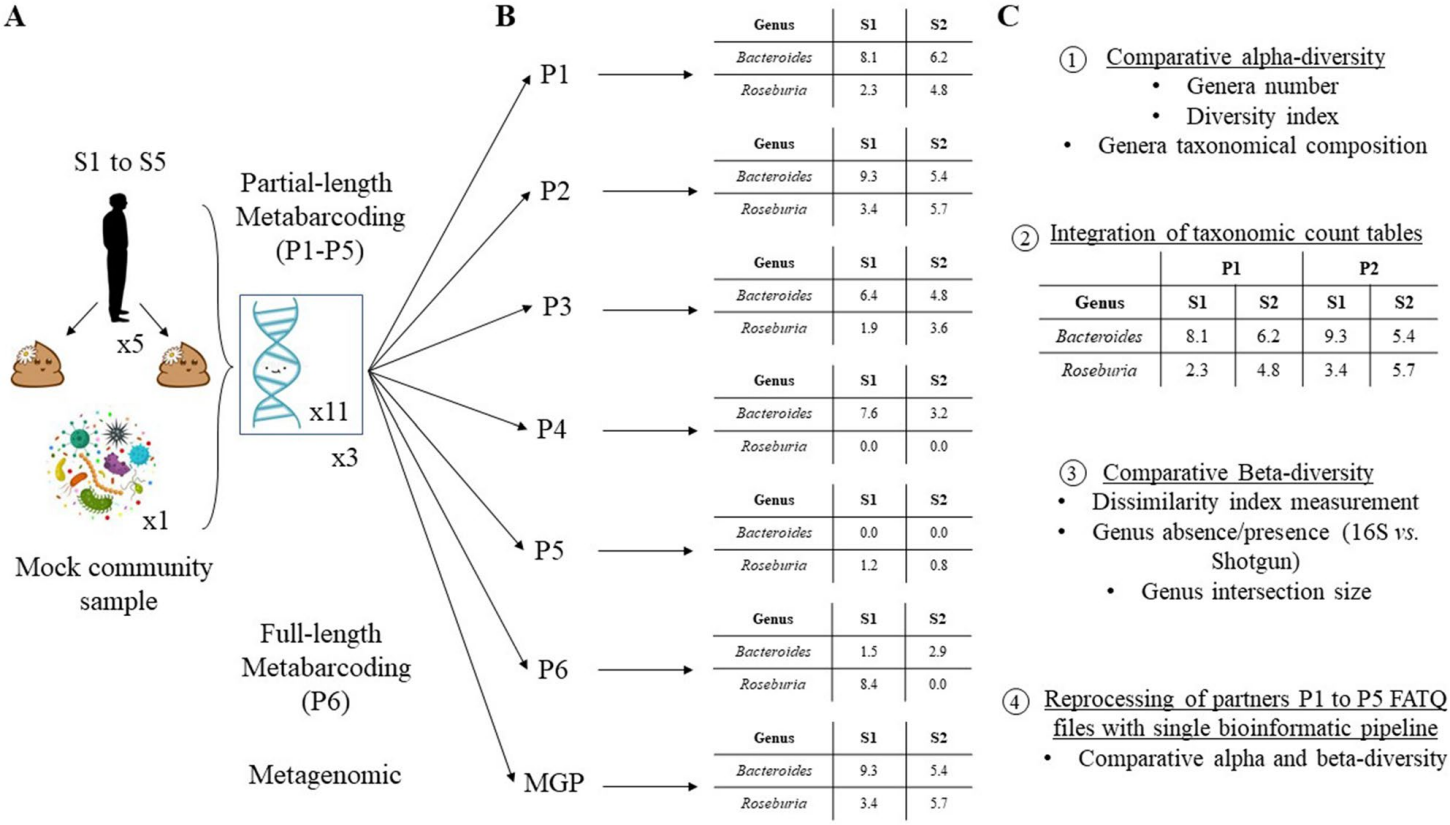
Schematic representation of the main sample preparation steps and follow-up analysis of the multi-center evaluation study of gut microbiome profiling. The study design consisted of three parts: (**A**) Consideration of five healthy human (S1 to S5) fecal samples collected at two time points and DNA extraction following IHMS standard protocols (http://www.human-microbiome.org/) as well as consideration of a microbial community DNA standard sample. (**B**) All DNA samples were provided in triplicates to five partners (P1 to P5) for partial-length metabarcoding sequencing and one partner for full-length metabarcoding using LUMI-Seq® (P6) as well as one partner for metagenomic DNA sequencing, following laboratory routine protocols. The genera *Bacteroides* and *Roseburia* were chosen as examples (**C**) Sample alpha- and beta-diversity analysis of bacterial genus profiles provided by each partner. Reprocessing of P1 to P5 sequencing data and alpha- and beta-diversity analysis performed on the bacterial genus profile.

Five healthy adult subjects (S1–S5) were enrolled. All research was performed in accordance with guidelines approval for the Institut Nationale de Recherche pour l'Agriculture, l'Alimentation et l'Environnement (INRAE) to manage human-derived biological samples granted by the Ministry of Research and Education under approval number DC-2012-1728. Informed consent was obtained from all five donors. Their fecal samples were collected at two time points in October 2018 (Sx_1) and January 2019 (Sx_2) following the IHMS protocols (SOP 05_V2) (Fig. 1A). The samples were collected by the subjects at home and stored at room temperature in a stabilizing solution (RNAlater® Stabilization Solution, Thermo Fisher Scientific, Waltham, USA), and transported within 24 h to our facilities.

### DNA extraction, standard solution, and transfer to partner laboratories

DNA extraction from stool samples was performed as recommended by IHMS SOP 06_V2 (MGP SOP 06_V3), in triplicate using a QIAsymphony® DSP Virus/Pathogen Midi Kit (Qiagen). ZymoBIOMICS™ Microbial Community DNA Standard was used as an internal positive control (ref. D6306; Zymo Research) (Fig. 1A). It contains a mixture of genomic DNA extracted from 10 microbial species comprising 8 bacteria (12% genomic DNA abundance each), a yeast, and a protist (2% genomic DNA abundance each), altogether covering a wide range of GC contents (from 15 to 85%). Before further processing, the extracted DNA was quantified using Qubit™ Fluorometric Quantitation (Qubit™ dsDNA HS kit, ref Q32851, Thermo Fisher Scientific) and qualified using DNA size profiling on a Fragment Analyzer™ (Genomic DNA 50 kb kit, ref DNF-467-O500, Agilent Technologies, Santa Clara, USA). Multiple DNA extracts obtained from similar fecal samples were pooled and mix as a unique solution that was shared in equal amount among partner laboratories. Partner laboratories (P1 to P6) received 1 µg of the DNA extracted in triplicate from the 10 fecal samples as well as triplicate solutions of the mock community DNA. Partner laboratories were not able to visually differentiate mock community DNA from human fecal DNA samples, allowing us to use this mock community DNA as internal control. The transfer was done in a box by mail in 2 mL Eppendorf tubes maintained at 4 °C using cold packs.

### Metagenomic and metabarcoding DNA sequencing

Metagenomic analysis was performed in MetaGenoPolis unit at the INRAE facilities by shotgun DNA sequencing (Fig. 1B, MGP). The libraries were generated from 1 µg of high-molecular-weight DNA (> 20 kbp). Shearing of the DNA into fragments of approximately 150 bp was performed using an ultrasonicator E220 system (Covaris, Woburn, USA), and DNA fragment library construction was performed using the 5500 SOLiD™ Fragment 48 Library Core Kit (Thermo Fisher Scientific). Purified and amplified DNA fragment libraries were sequenced using an Ion Proton™ Sequencer (Thermo Fisher Scientific), with a minimum of 20 million high-quality single-end reads (150 bp) per library.

Metabarcoding sequencing was performed in the partner laboratories (P1 to P6). P1 to P5 performed partial-length (Fig. 1B, P1 to P5) and P6 full-length metabarcoding sequencing of the 16S rRNA gene (Fig. 1B, P6).

Details of the metabarcoding materials and methods used by each partner are provided in Supplementary Table 1. Upon reception, all partners performed DNA quantity control, P1 and P2 additionally performed DNA purity control, which also included DNA size control using dedicated methodologies. P1 to P5 sequenced the V3–V4 regions of the 16S rRNA gene 2 × 250 bp, or 2 × 300 bp for P2, using a MiSeq sequencer (Illumina, San Diego, USA). P1 and P2 used the same pair of primers, while the other partners such as P3, P4, and P5 used different primer pairs but with few differences (Supplementary Table 2). P6 built libraries using the LUMI-Seq® methodology, enabling the sequencing of full-length 16S rRNA genes using the Illumina short-read platform. The method incorporated randomized unique molecular barcodes on the 5ʹ ends of individual 16S rRNA gene template molecules. After molecular barcoding, the full-length 16S gene V1–V9 was PCR amplified in order to make multiple copies and to increase the signal. The full-length fragments were then enzymatically tagmented while keeping the UMI tag on all pieces. The library was then sequenced using a NextSeq500 sequencer, in 2 × 150 bp, using the classical Illumina sequencing workflow. Each partner committed to produce a minimum of 40 k reads per DNA sample. Following sequencing, the number of total raw reads per partial-length metabarcoding partner varied from 3.64 (P3) to 18.99 (P5) million reads (Supplementary Fig. 1, raw reads number). P6 sequenced an average of 6,625 full-length 16S rRNA reads per sample.

### Bioinformatic data analysis

Metagenomic reads were cleaned using Alien Trimmer v0.2.4^37^ to remove resilient sequencing adapters and trim low-quality nucleotides at the 3ʹ side using a quality and length cut-off of 20 and 45 bp, respectively. Cleaned reads were subsequently filtered from human and other possible food contaminant DNA (using human genome RCh37-p10, *Bos taurus,* and *Arabidopsis thaliana* with an identity score threshold of 97%). Filtered high-quality reads were mapped with an identity threshold of 95% upon mapping on the 10.4 million genes Integrated Gut Catalogue 2 (IGC2)^38^, using Bowtie v2.2.6^39^ included in METEOR v3.2 software^40^. A table of the gene abundance was generated by means of a two-step procedure using METEOR. First, the unique mapped reads (reads mapped to a unique gene in the catalogue) were attributed to their corresponding genes. Second, shared reads (reads that mapped with the same alignment score to multiple genes in the catalogue) were attributed according to the ratio of their unique mapping counts. The gene abundance table was processed for rarefaction and normalization and further analysis using the MetaOMineR v1.2 (momr) R package^41^. To decrease technical bias due to different sequencing depths and avoid any artifacts of sample size on low-abundance genes, read counts were rarefied. The gene abundance table was rarefied to 14 million reads per sample by random sampling and removing without replacement. The resulting rarefied gene abundance table was normalized according to the FPKM strategy (normalization by the gene size and the number of total mapped reads reported in frequency) to generate the gene abundance profile table. The gene count was computed as the number of genes detected (i.e., with a strictly positive abundance) in a given sample after downsizing. For taxonomic profiling, the IGC2 catalogue was organized into 1990 Metagenomic Species (MSP), i.e., clusters with a minimum of 100 genes, using MSPminer^42^. MSP taxonomy was assigned with the Genome Taxonomy Database^43^. The relative abundance of an MSP was computed as the mean abundance of its 100 ‘marker’ genes (i.e., the genes that correlate the most altogether). If less than 10% of ‘marker’ genes were seen in a sample, the abundance of the MSP was set to 0. The relative abundances at higher taxonomical ranks were computed as the sum of the relative abundances of the MSP that belong to a given taxon. The MSP count was assessed as the number of MSP present in a sample (i.e., with a strictly positive abundance). The MSP table was then resolved at the genus taxonomical level. For DNA sequences obtained from mock community samples, genomes of microbial strains present in the reagent and downloaded from a link specified in the instruction manual of the provider (ZymoResearch) were considered to build a catalogue that was further used as a reference database to obtain relative abundances of microbial species using Kraken v.2.1.0^44^. We acknowledge that the choice to use a genome catalogue of the ten species present in the mock community, according to the manufacturer’s recommendation, makes it impossible to identify other species.

The key steps of the bioinformatic pipeline used to analyze the metabarcoding data are described in Supplementary Table 2. P1 and P5 used mothur^45^ as the data analysis software, while P3 and P4 used QIIME^46^, P2 used FROGS^47^ and P6 DADA2^48^. Only DADA2 used ASV as the clustering strategy, while the others used OTU. Among the partners using OTU clustering, we noticed the use of different software, except for P1 and P5, who used a similar one, OptiClust^49^. P2 used Swarm^50^, P3 used UCLUST^51^, P4 used USEARCH^51^. OptiClust, UCLUST, and USEARCH use a clustering approach based on centroid selection and a global clustering threshold (set to 97% similarity), where closely related amplicons can be placed into different OTUs, while Swarm clusters iteratively by using a small user-chosen local clustering threshold, allowing OTUs to reach their natural limits^49^. All partners used FastQC for read quality control. The partners used different read merging software, except P1 and P3, who used FLASH^52^. Following reads merging, we noticed that P1 obtain merged reads shorter (~ 150 bp) than those obtained by the other partners (~ 450 bp). Different thresholds were used for sequence removal (Supplementary Table 1). P1, P3, and P5 removed chimeric sequences, P2, P4, and P5 removed rare OTUs, P2 and P6 removed sequences based on read size, P3 based on read quality, and P1 was the only partner removing homopolymers. P3 and P4 did not use sequence denoising software. Except for P3 and P4, who used the same RDP classifier tools^53^, the other partners used different taxonomic affiliation methods. P5 used Greengenes^54^, P2 used NCBI Blast + ^55^, and P6 a naive Bayesian classifier^56^. For partial-length metabarcoding, three different reference databases for taxonomic annotation were used. P1 and P3 used Greengenes^54^, P2 and P4 used SILVA^57^, and P5 used RDP. P2 returned all taxonomies considering all blast best hits and a consensus taxonomy with tagging of ambiguous taxons as “Multi-affiliation”, which were considered as unclassified taxons in the following analysis. P6 using full-length metabarcoding used an in-house database. Bacterial and archaeal genomes at all assembly levels (Complete, Chromosome, Scaffold, Contig) were downloaded from the RefSeq database^58^ in May 2019, using ncbi-genome-download version 0.2.9^59^. The taxonomic information of the genomes were retrieved from the NCBI Taxonomy database taxdump files downloaded from the Taxonomy FTP site^60^. The 16S rRNA sequences were extracted from the genomes using barrnap version 0.9^61^. For each genome, the extracted sequences were compared with each other using the clustering tool CD-HIT version 3.1^62,63^ to keep only the unique sequences in each genome. The database was complemented by a curated collection of prokaryotic 16S rRNA sequences from the 16S rRNA RefSeq Targeted Loci Project^64^ download in April 2019. Sequences containing non-standard nucleotides or having unclear species identity (e.g., containing “sp.”) were removed. All the remaining sequences were then clustered at 100% using CD-HIT. For each cluster, the longest sequence was defined as the representative sequence and the taxonomy of the sequences making up the cluster was checked if there is a majority (with a threshold of 90%). The majority taxonomy would be selected as the cluster annotation. Otherwise, the cluster annotation would move up the taxonomic rank. For example, if 50% of the sequences in a cluster are annotated to the species *Staphylococcus epidermidis* and 50% annotated to *Staphylococcus aureus*, we will go up to the genus level by assigning Staphylococcus to the cluster, leaving the annotation of the species level empty. The final database contains 72,954 sequences that represent 15,041 species, 3151 genera, 510 families, and 52 phyla. Following data trimming using a dedicated metabarcoding partner bioinformatic protocol, the number of sequences obtained by the partners varied from 1.7 to 8.2 million sequences (Supplementary Fig. 1, trimmed read). Sequence trimming removed more than 43% of the generated raw sequences in P2, which used stricter trimming conditions (read size, chimeric sequences, and rare OTUs) compared to the other partners such as P1 and P5, with 25% of the original raw sequences removed and P3 and P4 with less than 2%.

In order to measure biases with partial-length metabarcoding partners due to the bioinformatics pipeline only, the data analysis was repeated from demultiplexed raw sequence data provided by P1 to P5, using a single and new bioinformatics pipeline. The key steps of the bioinformatics pipeline used to analyze the metabarcoding data are described in Supplementary Table 3. Briefly, we used cutadapt^65^ to remove primers and SPAdes^66^ to correct for sequencing errors. The paired-end reads were merged using PEAR^67^, chimeric sequences were removed using UCHIME 3^68^, and the remaining sequences were clustered into ASV using Vsearch (v2.15.1). Importantly, ASVs with numbers of sequences below 8 counts were removed (default parameter). For phylogeny identification, RDPTools suit v2.11^69^ was used. Following data trimming, approximately 30% of original raw sequences were removed for P5, which is in a similar range as what the partner previously obtained, and 50% for P1, P2, and P3, which is two times higher for P1 and twenty-five times higher than P3 compared to what the partners previously obtained and in a similar range for P2. More surprisingly, 85% of the raw sequences generated by P4 were removed (Supplementary Fig. 1, reprocess trimmed read) when less than 2% of the sequences were removed by this partner.

### Statistical and data analysis

The total number of bacterial genera identified in all samples, by each partner, was summed to calculate the genus richness. This count was performed in case genera were present in at least two replicates out of three. The partners provided the Shannon diversity index, calculated at the species taxonomic or OTU level, as a measure of the alpha-diversity. Both measurements of alpha-diversity were used to draw the boxplot visualization carried out with the *ggplot* function from the ggplot2 R package. Venn diagrams were plotted using the *draw.pairwise.venn* function of the VennDiagram R package. The bacterial genera relative abundance count tables provided by the partners were concatenated into a single table (Fig. 1C). A list of all bacterial genera provided by the partners’ relative count tables was made into a single entity and then re-associated with relative abundances provided by individual partners using the VLOOKUP function in Excel. Bray–Curtis indexes were calculated using the *vegdist* function of the vegan R package as a measure of the beta-diversity. Stacked bar plots with hierarchical clustering for visualization in a dendrogram form were drawn based on the Bray–Curtis index values, using the *as.dendogram, upgma* and *gglplot* functions of the ggplot2 R package. PCoA (principal coordinate analysis) visualizations were carried out using the *pcoa* and *s.class* functions of the ade4 and ape R package from Bray–Curtis dissimilarity matrices. Common and partner-specific bacterial genera were visualized using *UpSet* plots with the UpSet R package. All statistical analyses were performed using R v.4.1.2 software (http://cran.r-project.org/). For phylogenetic tree visualization, 16S rRNA genes sequence alignment was carried out with *ClustalOmega* (using default parameters), and the alignment files were then submitted to a phylogenetic analysis using Phylogeny.fr customized workflow service^70^ including alignment curation with *Gblocks* (using default parameters)^71^, tree construction with *PhyML* (boostrap 100)^72^, and visualization by *TreeDyn*^73^.

### Ethical approval and consent to participate

Each donor consent to participate in the protocol signing consent form and accepting their samples to be conserve in our biobank. Approval for the Institut Nationale de Recherche pour l'Agriculture, l'Alimentation et l'Environnement (INRAE) to manage human-derived biological samples was granted by the Ministry of Research and Education under approval number DC-2012-1728, updated DC-2020-1728.

## Results

### Bacterial profile variations in mock communities at the genus level

**Figure 2:**
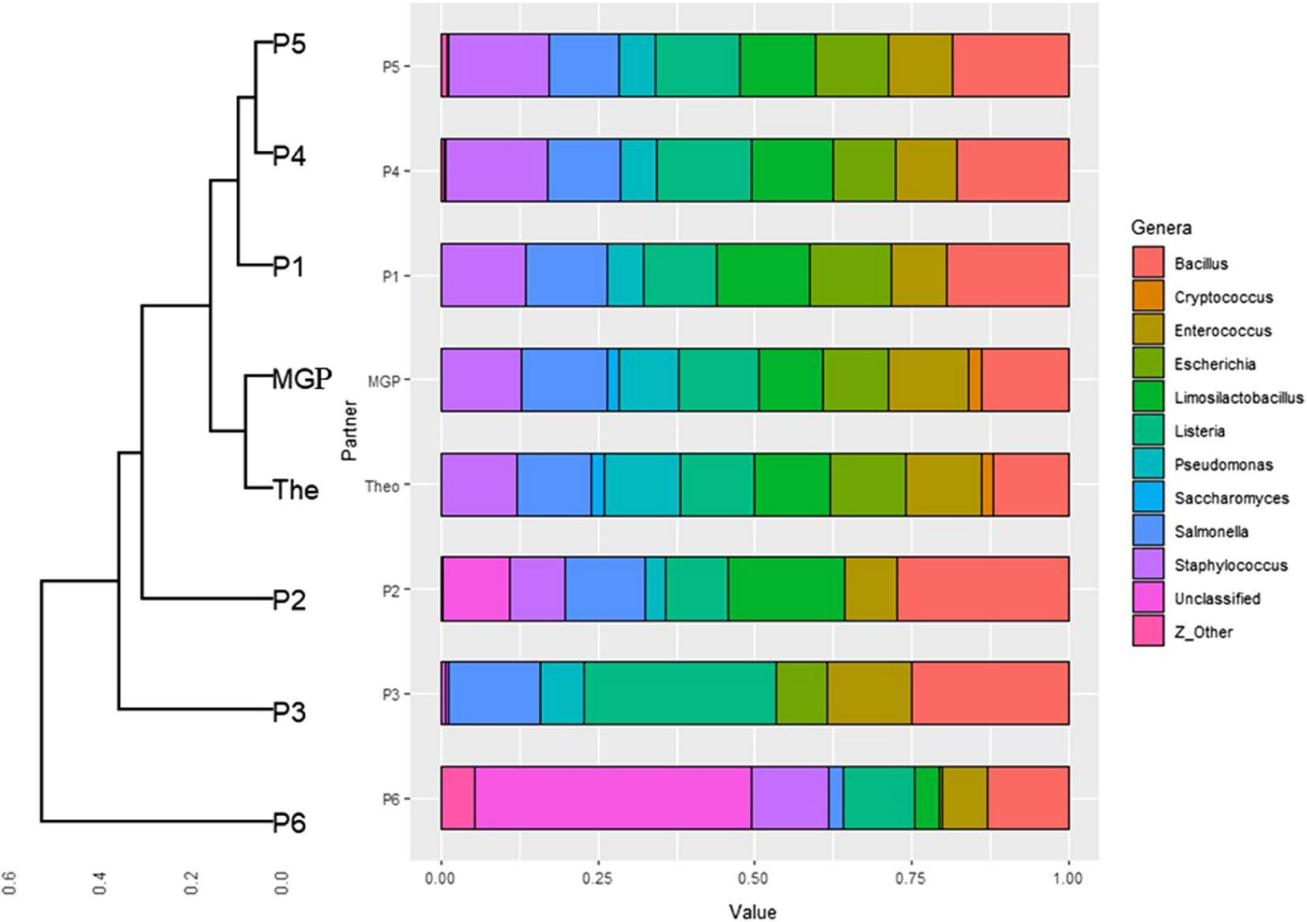
Stacked bar plot with hierarchical clustering (dendrogram), based on calculation of the Bray–Curtis dissimilarity index of mock samples. Microbial genera relative abundance is averaged from triplicate samples obtained by shotgun (MGP) and 16S rRNA (P1 to P6) sequencing strategy and compared to the theoretical profile (The).

While 16S rRNA metabarcoding only identified bacteria and archaea, *Saccharomyces cerevisiae* and *Cryptosporidium* were only identified by a shotgun metagenomics approach. Only three metabarcoding partners (P1, P4, and P5), out of six, detected all eight bacterial species present in the mock sample. Partner P2 missed *Escherichia*, P3 missed *Limosilactobacillus*, and P6 with LUMI-Seq® missed *Pseudomonas*. P2 and P6 had the highest count of unclassified genera, 10.8% ± 0.4 and 44.2% ± 0.6, respectively. The high proportion of unclassified genera identified by P6 led to underestimation of *Escherichia*, *Limosilactobacillus*, and *Salmonella* abundances. P2 and P3 overestimated the relative proportion of *Bacillus* and underestimated the proportion of *Pseudomonas*. P3 overestimated the relative proportion of *Listeria* and underestimated the relative abundance of *Staphylococcus*. Overall, from beta-diversity analysis based on Bray–Curtis dissimilarity indexes (Fig. 2), MGP provided the best proximity with the theoretical profile, this result being expected as identification of other species was not possible, followed by P1, P4, and P5, while P2, P3, and P6 stood out as outliers. Thus, beta-diversity analysis highlighted the lower ability of P2, P3, and P6 to correctly profile the reference sample at the bacterial genus level as well as the good performance of P1.

### Comparative alpha-diversity and genus richness analysis in mock and human fecal samples

**Figure 3:**
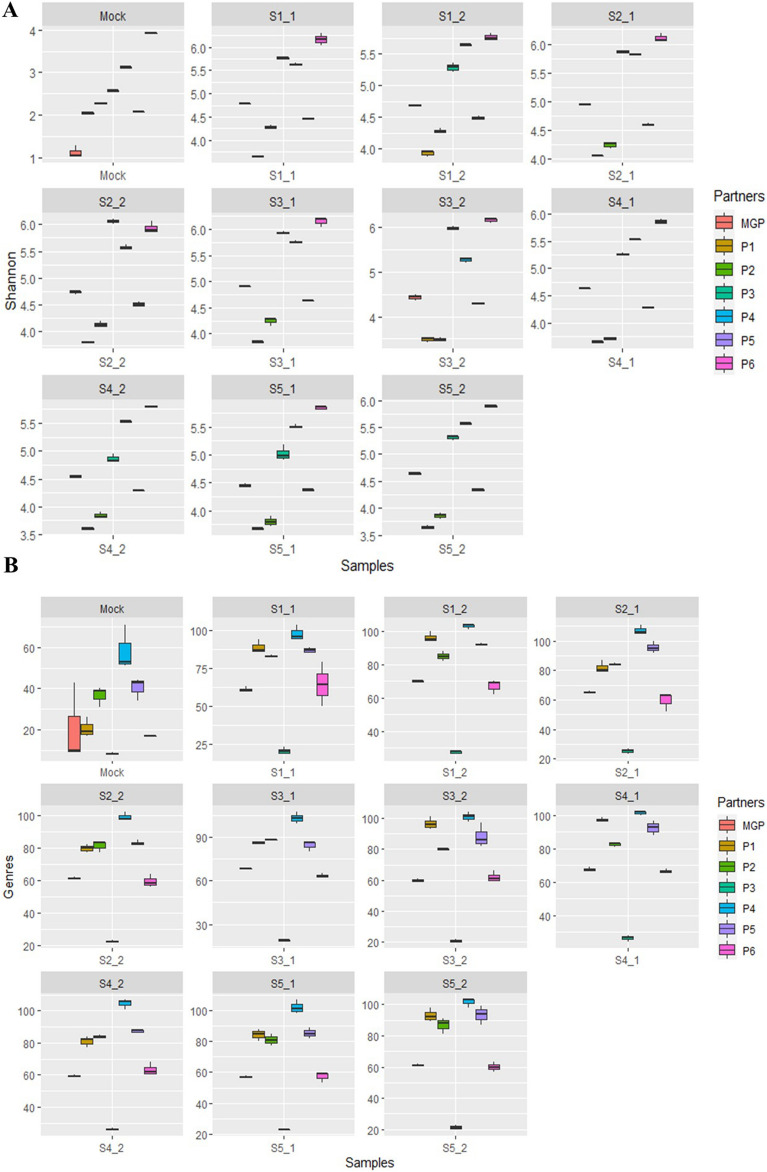
Boxplots of the alpha-diversity comparative analysis base on microbial genus number identified in samples for all partners (MGP, P1 to P6). (**A**) Average Shannon index calculated by partners on the basis of bacterial species diversity. (**B**) Average number of bacterial genera identified per triplicate sample with the standard deviation.

In mock sample, all metabarcoding partners overestimated the alpha-diversity, due to the identification of additional bacterial species, compared to the theoretical value (Fig. 3A). For the human fecal samples, P1, P2, and P5 underestimated while P3, P4, and P6 overestimated the alpha-diversity compared to the indexes calculated by MGP. P1 and P6 were outliers in their respective groups. For the human fecal samples, we noticed a substantial degree of inter-metabarcoding partner variation for genus richness measured from identical DNA samples, as for samples S3_1, partner P3 identified an average of 18.7 ± 0.6 genera while P4 identified 103 ± 4 genera (Fig. 3B). Overall, for any sample considered, the difference in the bacterial genus richness between P3 and P4 was the highest. P1, P2, P4, and P5 overestimated the bacterial genus richness compared to MGP and P6, who identified comparable genus richness, while it was underestimated by partner P3.

While all partners identified unclassified bacterial genera, their average relative abundances varied from 7.9 for P4 to 61.9% for P6 (Supplementary Fig. 2A). Among the partial-length metabarcoding partners, P1, P2, and P4 had the lowest average relative abundances of unclassified bacterial genera, ranging from 7.9% for P4 to 21.9% for P2, followed by P3 and P5 with 47.7% and 34%, respectively. Unexpectedly, these proportions of unclassified bacterial genera among partial-length 16S rRNA were lower than those obtained by laboratories using higher sequencing taxonomical resolution such as MGP or full-length sequencing using LUMI-Seq®, with 51.4% and 61.9%, respectively. Thus, P1, P2, and P4 were using weaker conditions for classification of OTU clusters compared to the other partners.

By contrast, with a performance similar to those obtained for bacterial genus profiling using mock samples, P3 missed the most abundant human gut genera such as *Bacteroides, Parabacteroides*, and *Prevotella*, which were not detected or identified at low relative abundance (e.g., *Faecalibacterium* (< 0.05%)—Supplementary Fig. 3). These bacterial genera are core members of the human gut microbiota, and analytical pipelines missing them may be identified and flagged as poor service providers in gut microbiota analysis by regulatory and legal agencies.

### Comparative beta-diversity analysis in human fecal samples

**Figure 4:**
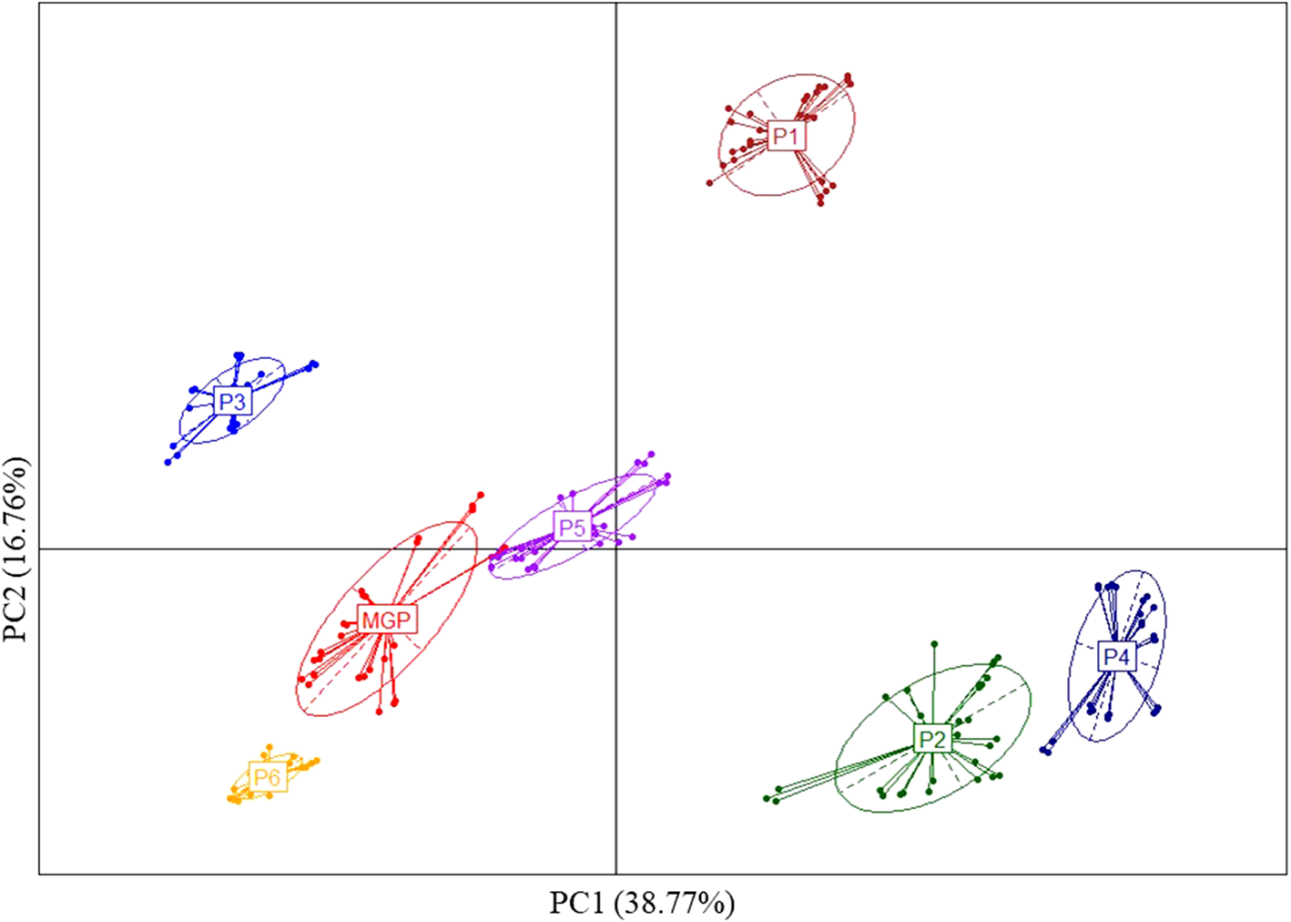
Scatter plot of the two first principal components obtain using PCoA based on Bray–Curtis dissimilarity indexes calculated between the genus bacterial profiles. Beta-diversity analysis of the microbiota profiles at the genus level and provided by the partners (P1-P6) and MGP.

Following the aggregation of counting tables, at the bacterial genus level, provided by the partners after sequencing of DNA isolated from stool samples, a total of 429 unique genera were identified. For human fecal samples, the dissimilarity between all pairs of partners, as measured by Bray–Curtis indexes at the bacterial genus level, was such that we observed lower inter-individual variance than inter-partner variance (Fig. 4). The microbiota profiles depicted by partners P5 and P6 were the most similar to those obtained by MGP. P2 and P4, which clustered together, as well as the P3 and P1 partners, provided microbiota profiles that were the most dissimilar to those of the other partners. We also noticed that this clustering was markedly influenced by the taxonomic database that was used for the phylogenetic annotation (Supplementary Table 1). The number of confounding factors exceeding the number of laboratories, all conclusions should be taken with caution.

Considering bacterial genera identified as shared between the metabarcoding partners and MGP or exclusively identified by a single partner (Supplementary Fig. 4A), we observed three groups of partners. The first cluster comprised P1, P2, and P4, for which most of the identified bacterial genera were only identified by metabarcoding. The average total relative abundance of these partner-exclusive bacterial genera ranged from 10.6 to 29.8% for P1 and 30.8 to 65% for P2 and P4, with a high variance between samples. For P1 and P4, these results confirm their use of weaker parameters for OTU classification at the genus level, as described earlier. The second cluster comprised P3, for which most of the identified bacterial genera were only identified by MGP, and these bacterial genera represented an average total relative abundance between 20.1 and 28.4%, depending on the samples. This confirms the tendency of P3 to miss bacterial genera classification. The third cluster, comprising P5 and P6, had most of its identified bacterial genera shared between the partners and MGP, which explained their similarity as measured by the Bray–Curtis distance. For the two latter partners (P5 and P6), the bacterial genera identified by MGP only represented a low average total relative abundance, ranging from 2.2 to 8.2% for P5 and 0.3% to 5.7% for P6. Overall, the bacterial genera exclusively identified by the metabarcoding partners represented a higher average total relative abundance compared to those exclusively identified by MGP.

**Figure 5:**
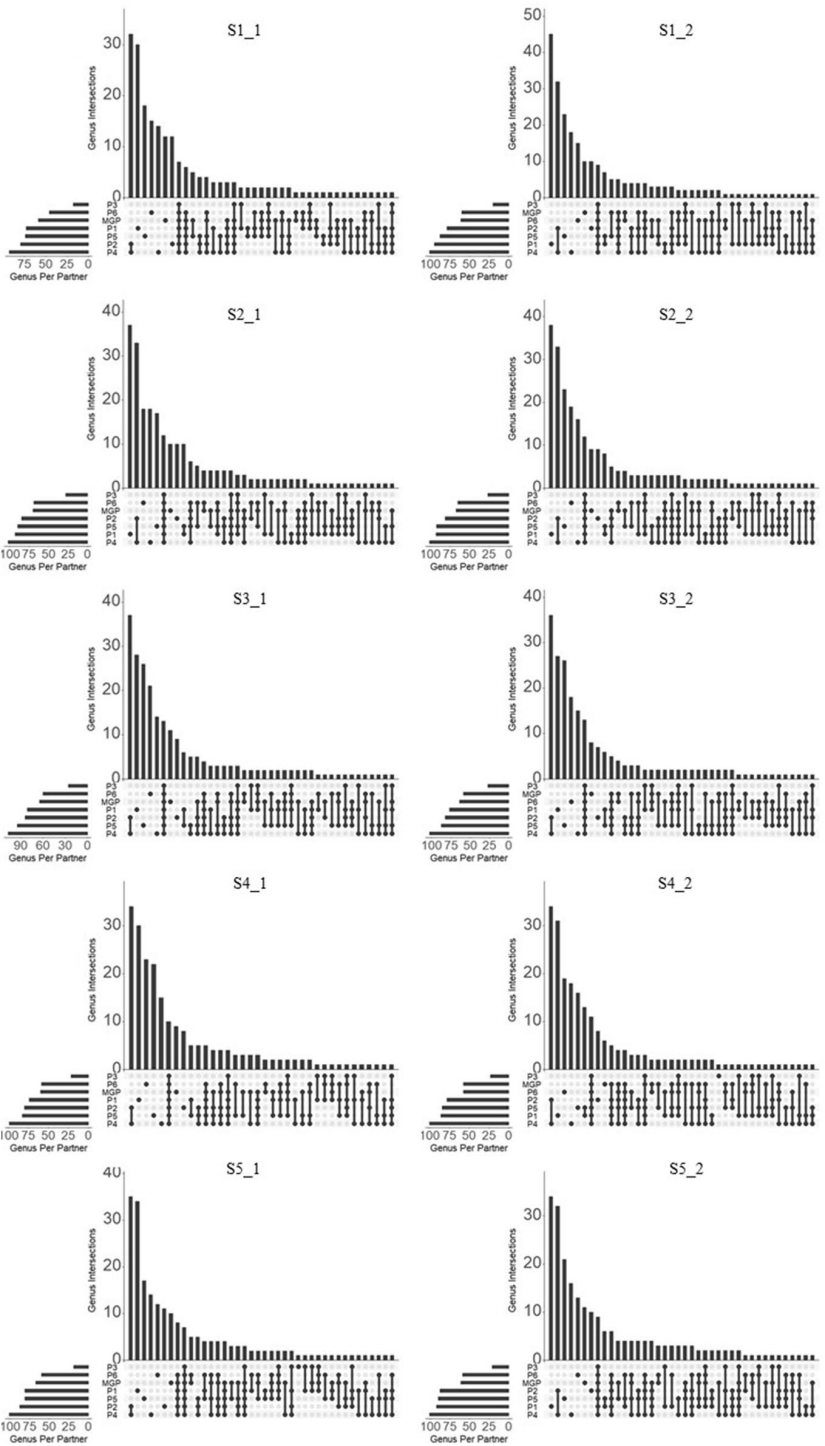
UpSet plot representing number of bacterial genera in the human fecal samples (S1_1 to S5_2) exclusively identified by one partner or shared between multiple partners (MGP, P1, P2, P3, P4, P5, and P6).

In a genus intersection count analysis performed between all partners (Fig. 5), P1 had the highest number of genera exclusively identified by a single partner, between 27 and 45, which accounted for approximately 34.6% (S3_1) to 47.4% (S1_2) of the total number of genera identified by this partner and represented between 2.9 and 10.2% of the total relative abundances of the bacterial genera identified by this partner. P1 thus accounted for a total of 159 partner-exclusive bacterial genera, representing a low proportion of the total relative abundance (Supplementary Table 4). The tendency of P1 to identify such a high number of partner-exclusive bacterial genera contributes to the high genus richness and β-diversity dissimilarity measured compared with the other partners (Figs. 3B and 4). On the other hand, P3 did not identify any partner-exclusive bacterial genera, except one in S4_2. This observation confirms the low genus richness previously reported (Fig. 3B) as well as the tendency of this partner to misidentify the core gut bacterial genera. In most samples, P5, P6, and P4 identified between 12 and 26 partner-exclusive bacterial genera, which is higher than MGP, and P2, which identified 10 partner-exclusive bacterial genera. P2 and P4 shared the highest number of bacterial genera (32 to 37), varying between 24.3 and 60.3% of the total relative abundances of the bacterial genera identified by these partners. P2 and P4 thus accounted for a total of 43 partner-exclusive bacterial genera, representing a high proportion of the total relative abundance (Supplementary Table 5). We noticed that most of the exclusive bacterial genera common to P2 and P4 were different taxonomic sub-divisions of genera, such as *Ruminococcus* sub-divided in Ruminococcaceae *NK4A214* or *UCG-002*, *-003*, *-004*, *-005*, *-009*, *-010*, *-013*, *-014*, *Ruminococcus 1* and *2* or *Prevotella*, *Ruminiclostridium, or Lachnospira*. All these taxonomic intermediate names present in the SILVA database used by both partners (Supplementary Table 1) largely contributed to the β-diversity similarity of the bacterial genera profiles observed between P2 and P4 and their dissimilarity with the other partners (Fig. 4). This also explains why P2 and P4 overestimated the bacterial genus richness. However, these sequences associated with genera sub-groups or intermediate taxonomic ranks mainly corresponded to yet uncultured bacterial groups and were not well defined at the genus level (Supplementary Fig. 5). Partner P5 identified between 14 and 22 partner-exclusive bacterial genera, which represented between 1.5 and 8.1% of the total diversity. P6 identified between 12 and 21 partner-exclusive bacterial genera, which represented between 4.9 and 9.7% of the total diversity. MGP identified between 6 and 14 partner-exclusive bacterial genera, which represented between 0.5 and 6.7% of the total diversity. This relatively low amount of partner-exclusive bacterial genera accounting for a low total relative abundance contributed to the low dissimilarity of the bacterial genera profile as measured by β-diversity analysis between P5, P6, and MGP (Fig. 4). The number of shared bacterial genera among all partners was between 7 and 15, depending on the samples, representing from 3.1 to 34.8% of the total relative abundances of bacterial genera, depending on the partners.

A summary of the criteria used for classification by the metabarcoding partners based on their capacity to approximate bacterial genus profile as measured by the use of metagenomic is presented (Table 1).

**Table 1:** Summary table of the metabarcoding partners’ individual performances compared to the results obtained in shotgun metagenomic bacterial profiling based on genus presence/absence, α- and β-diversity indexes. 0: poor performer, 1: medium performer, 2: good performer.

Partners	Mock				Feces				Total scoring	Rank	Remarks
Criteria	Genus presence/absence	α-diversity		β-diversity	Genus presence/absence	α-diversity		β-diversity			
		Shannon index	Genus richness			Shannon index	Genus richness				
P1	2	2	2	2	0	0	1	0	9	2nd	Despite good performance to profile mock bacterial community samples, P1 identifies too high a of number of partner-exclusive bacterial genera at low abundances, which may be explained by the use of thresholds that are too low for genus-level sequence assignment for taxonomic annotation and/or outdated database, as Greengenes, for taxonomicannotation
P2	0	2	1	0	1	1	1	0	6	5th	Counting too high of a proportion of unclassified sequences in mock bacterial community samples, missing Escherichia, P2 also identifies too high of a proportion of partner-exclusive bacterial genera, which may be explain by use the taxonomic database SILVA, accounting for numerous sub-genera or intermediate genus taxonomical groups
P3	0	1	2	0	0	2	0	0	5	6th	Failing to identify numerous important members of the mock bacterial community and gut microbiota, P3 also provided the lowest genus richness and the highest proportion of unclassified sequences. Bacterial profiling remains as an outlier following use of a single bioinformatic pipeline. We make the assumption that the choice of primers for 16S rRNA amplification combined with the use of outdated database such as Greengenes greatly impacts the outcome of the analysis
P4	2	0	0	2	1	2	0	0	7	4th	Overestimates species diversity and genus richness, P4 also identifies a lower proportion of unclassified sequences, which may be due to the use of thresholds that are too low for genus-level sequence assignment for taxonomic annotation. P4 also identifies a higher proportion of partner-exclusive bacterial genera, which may be explain by use the taxonomic database SILVA, accounting for numerous sub-genera or intermediate genus taxonomical groups
**P5**	2	2	1	2	2	1	1	2	13	1st	Considering the bacterial profiles obtained compared to MGP, this partner performs the best compared to all other for almost all criteria expect the α-diversity, which appears to be slightly overestimated but representing a low proportion of bacterial genus relative abundance
P6	0	0	2	0	2	0	2	2	8	3rd	Despite a tendency for the identification of unclassified bacteria both in mock and fecal samples, which may be due to the scarcity of full-length 16S rRNA gene in the database used, P6 provides a good similarity with bacterial profiles as measured by shotgun metagenomics

### Comparative analysis of the bacterial profiles obtained after reanalysis of partial-length metabarcoding datasets

To measure the specific impact of bioinformatic pipelines on the bacterial genera profiles obtained from the partial-length metabarcoding partners (P1 to P5), we reanalyzed the raw sequence datasets issued from all partners with the use of a single bioinformatic pipeline (Supplementary Table 3).

For the mock samples, the bacterial genus profiles were similar to those obtained previously, except that P2 displayed a lower prevalence of unclassified genus and identified *Escherichia*. P3 still missed the *Limosilactobacillus* genus (data not shown). For the human fecal samples, the average relative abundances of the unclassified bacterial genera varied from 20.4 to 22% for P1, P2, P4, and P5 and 37.2% for P3 (Supplementary Fig. 2B). A higher proportion of the sequences provided by P5 was classified following the new analysis. Only the sequencing data provided by P3 had a high proportion of unclassified bacterial genera, while for the other metabarcoding sequencing partners the proportion of unclassified bacterial genera was rather uniform. The reprocessing of sequencing data identified a total of 180 bacterial genera, so less than half of those identified previously. Variation of the alpha-diversity, measured by the Shannon diversity index at the species level and genus richness, between the partners was lower than that measured previously. The average Shannon diversity index varied between 3.5 and 6 in the original datasets and between 4 and 5 upon reanalysis, depending on the partners (Supplementary Fig. 6A). The average genus richness varied between 23.2 and 102.1 in the original datasets, while the new assessments varied from 51.0 to 91.5, depending on the partners (Supplementary Fig. 6B). The bacterial species profiles obtained by P1 had the lowest average Shannon diversity index in the original datasets (3.7 ± 0.2). Following reanalysis, it had one of the highest average Shannon diversity indexes (4.8 ± 0.2). This increase in species alpha-diversity was also observed for P2 and P5, albeit to a lower extent. For P1 and P2, the reanalysis measured a lower richness at the genus level. The P1 and P2 partners had an increase in the bacterial species diversity index, which did not translate into an increase in the genus richness. In contrast, the bacterial species profiles obtained by P3 had the highest average Shannon diversity index in the original datasets (5.5 ± 0.4), whereas reanalysis led to the lowest average Shannon diversity index (4.3 ± 0.3). This decrease in species diversity following reanalysis was associated with an increase in the genus richness, which varied from 23.2 ± 3.1 to 50.9 ± 3.8. Thus, for this partner, a decrease in the bacterial species diversity index did not translate into a decreased genus richness. This decrease in the species alpha-diversity was also observed, albeit at a lower extent, for P4, which, in this case, also translated into a decreased genus richness upon reanalysis. The changes in the alpha-diversity index and genus richness for partner P5 were very small.

**Figure 6:**
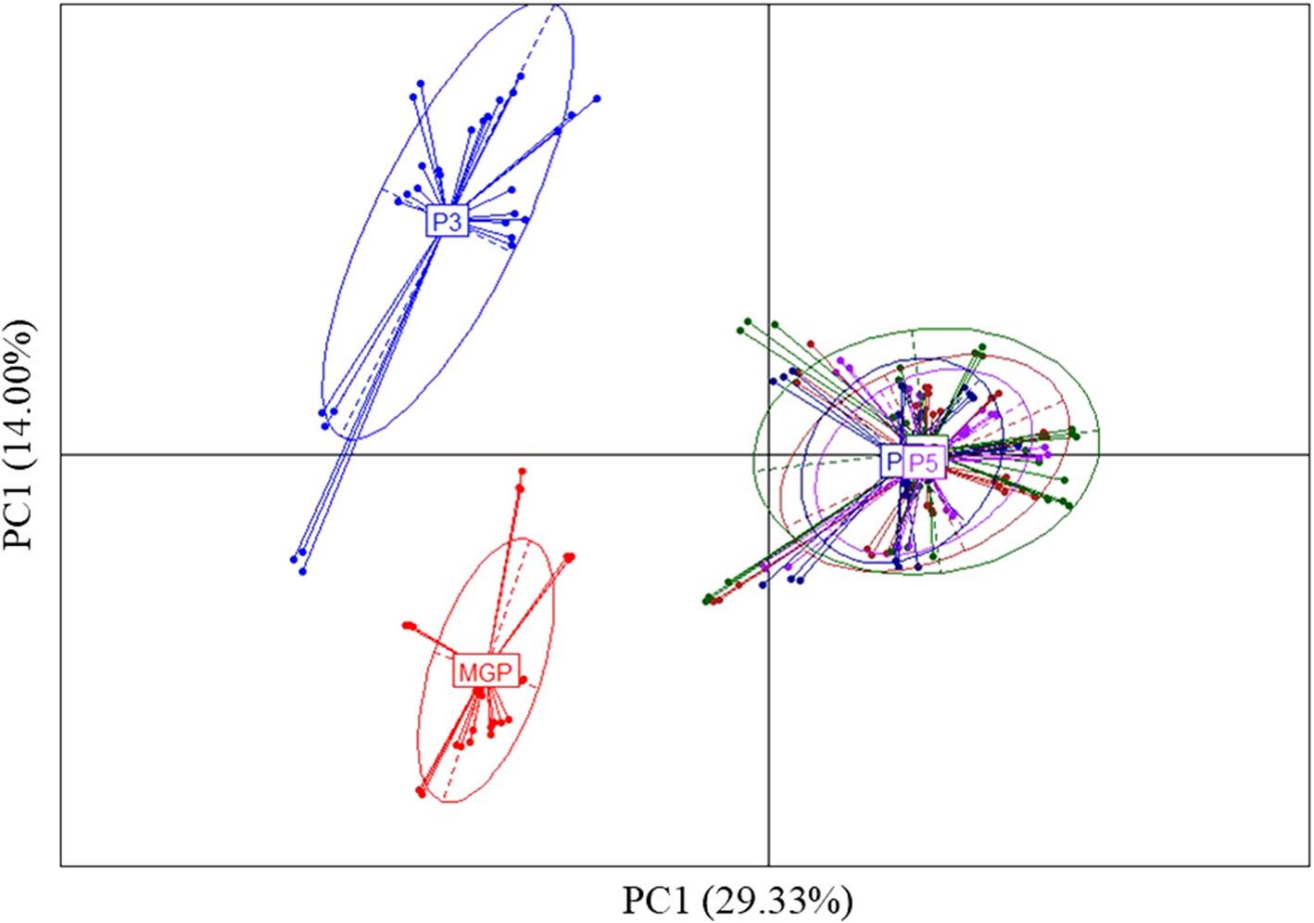
Scatter plot of the two first principal components obtain using PCoA based on Bray–Curtis dissimilarity indexes calculated between the genus bacterial profiles following reanalysis of the raw dataset with a single bioinformatic pipeline. Beta-diversity analysis between the 16S rRNA sequencing partners (P1-P6) and MGP.

Following reanalysis and measurement of the beta-diversity as defined by the Bray–Curtis index, we found a higher similarity of the profiles obtained at the genus level between P1, P2, P4, and P5, but P3 remained as an outlier (Fig. 6). However, considering comparative analysis between the metabarcoding and metagenomic partners, we still found lower inter-individual variance than inter-laboratory variances, as previously observed. Considering the richness of bacterial genus identified as shared between metabarcoding and metagenomic or exclusively identified by a single partner (Supplementary Fig. 4B), the first group of partners (P1, P2, and P4), previously identified as the one with the highest number of bacterial genera exclusively identified by a single metabarcoding partner, was now the group with the most abundant shared bacterial genera. The metabarcoding partners-exclusive bacterial genera represented an average total relative abundance ranging from 8.4 to 26.9%, depending on the partners and samples. The values were of the same order of magnitude as those previously measured for P1, but they were lower for P2 and P4. The second group, represented by P3, was still dominated by bacterial genera exclusively identified by MGP, but their average total relative abundance ranging between 13.9 and 25.1%, depending on the samples, was slightly decreased. Among these genera, we still noticed the absence of the most abundant human gut bacterial genera, such as *Bacteroides* and *Prevotella*. The third group, represented by P5, was still dominated by bacterial genera shared between the metabarcoding and metagenomic partners. For this partner, the bacterial genera exclusively identified by metagenomics were still low, representing an average total relative abundance ranging from 2.5 to 7.8%, depending on the samples. In this reanalysis, the number of bacterial genera shared between all partners was higher, between 21 and 33 (Supplementary Fig. 7), representing 20.4% to 62.1% of the total relative abundances of the bacterial genera, followed by the metabarcoding partners-exclusive genera, accounting for 16 to 22 members or 7.1% to 29.6% of the total relative abundances. We noticed that exclusive bacterial genera were dominant in P5 and MGP, with 8 to 19, representing less than 0.2% of the relative abundances, and 14 to 20 genera, representing between 2.4 and 7.8% of the relative abundances, respectively. The data reprocessing did not shift bacterial genus profiles obtained by P5 compared to the other partners. This may be due to the fact that the taxonomic annotation database used in this reprocessing analysis was the same as the one used by P5. All partners, excluding P3, tended to share between 6 and 10 genera, accounting for 6.3% to 45.4% of the total relative abundances.

## Discussion

In this multicenter study, a similar DNA standard solution or DNA isolated from human feces samples were provided in triplicates to partners who are experts in gut microbiome profiling by shotgun metagenomics or metabarcoding with the aim of comparing the impact of their routine sequencing methods and bioinformatic pipelines in resolving bacterial profiles at the genus and OTU, ASV, or species level. To differentiate the impact of sequencing protocols from bioinformatic strategies, all raw sequencing data from the partial-length metabarcoding partners was reanalyzed using a single bioinformatic pipeline. The reasonable expectation is that inter-individual (inter-sample) differences should be the primary driver of microbiome profile stratification. Our work shows that this is not the case for metabarcoding pipelines, hence questioning the possibility of standardization of 16S rRNA-based approaches. Our findings highlight significant differences in the bacterial species alpha-diversity index, and genus richness, as well as significant differences in the beta-diversity index showing a lower inter-individual variance than inter-laboratory variances. Our study also reports a dominance of genera exclusively identified by a single metabarcoding partner. These differences in the bacterial genus profiles between partners, quantifying methodological biases, are seldom documented and appear greater in magnitude compared to the perceived expectations and to what has been reported to date in the literature.

The sequencing strategy, including the choice of primers for metabarcoding, greatly affects the analysis outcomes. P3 failed to identify a few common bacterial genera, such as *Limosilactobacillus* or the predominant human gut commensals *Bacteroides* or *Prevotella*, or identified them but at a too low proportion, such as *Faecalibacterium*, even following data reprocessing with a single and different bioinformatic pipeline. In the original dataset, while providing a high species α-diversity index, this partner is also the one with the lowest genus richness compared to the others. It has been claimed on the USEARCH website (https://drive5.com/usearch/manual/uclust_algo.html) that UCLUST, which was the clustering method used by this partner, is not designed for OTU clustering, and such observation has also been reported in an empirical study and may explain such inflating α-diversity. Following data reprocessing, these two alpha-diversity indices became among the lowest and more consistent with each other. This partner, providing the lowest number of raw sequences, also accounted for the highest proportion of unclassified sequences compared to the other partial-length metabarcoding partners, which is still impactful upon data reprocessing. Unlike the other partial-length metabarcoding partners, the change of bioinformatic pipeline does not allow correction of this outlier position highlighted by beta-diversity analysis of the original dataset. The absence or underestimation of a few of the most abundant human gut bacterial genera explains the outlier position of this partner and highlights the importance of the sequencing strategy. We can hypothesize that the primers and/or the PCR amplification protocol chosen by P3 do not amplify the V3-V4 region of the 16S rRNA gene of these bacterial genera.

The settings for genus-level sequence assignment will matter greatly in the final outcome of metabarcoding analysis. P1 presents the particularity of identifying many partner-exclusive bacterial genera, for most at a low proportion, but that together accounted for up to 10% of the total bacterial diversity. These were not identified following reprocessing of the raw sequences with a new bioinformatic pipeline. We hypothesize that the sequence homology threshold for bacterial genus identification in the bioinformatics pipeline was set too low to allow accurate bacterial genus identification, hence resulting in over-estimation of the diversity.

The choice of the reference database markedly influenced the genus-level sequence assignment, and thereby the outcome of metabarcoding analysis. P1 and P3 both used the Greengenes database for taxonomic classification. Unlike other the databases used in this study, such as SILVA and RDP, Greengenes taxonomy is assigned based on automatic de novo 16S rRNA gene tree construction and rank mapping from other taxonomic sources, mainly the NCBI, which is not curated. Although still included in some metabarcoding analysis packages, such as QIIME, the database has not been updated for the past ten years^74^. Although the Greengenes database website recommends use of more updated sources for taxonomical annotation, this database is still used and referenced in more than a thousand publications each year. Greengenes2^75^, a reference tree that unifies genomic and 16S rRNA databases, recently published, should give a real chance of standardization across methods. In beta-diversity analysis, P2 and P4 displayed similar microbiota profiles, which may be partly due to use of a common database for taxonomic identification. The use of the SILVA database and the presence of numerous sub-genera or intermediate taxonomical groups explains the high amount of partner-exclusive bacterial genera identified for these two partners, accounting for almost half of the retrieved total bacterial genera diversity. Most of the observed differences were due to the identification of bacterial genera sub-taxa, belonging to *Ruminococcus*, *Prevotella*, *Ruminiclostridium,* or *Lachnospira*, which are mostly represented by uncultured bacteria. As previously reported, this observation may be due to the high amount of sequences present in the SILVA database, with few of them being associated with intermediate taxonomical ranks that are not present in other databases^76^. In this case, we assume that the presence of hypothetical sub-groups of bacterial genera in the reference database is the main driver of the dissimilarity measured. To a lesser extent, a similar trend was also observed for partner P5, using the RDP database. However, only the genus, *Clostridium,* was divided into different sub-genera or intermediate ranks, which impacted the measurement of beta-diversity compared to MGP. Use of the RDP database nevertheless allowed the highest level of similarity to be obtained to the bacterial profiles obtained by MGP or full-length metabarcoding.

Separating the impact of sequencing platforms and bioinformatic pipelines in metabarcoding analysis shows that both will influence the outcome. Although the analysis of sequences derived from various sequencing platforms using a single unique pipeline allowed greater similarities to be obtained and diminished the “laboratory-effect” (inter-laboratory differences), it still did not allow inter-platform differences to be completely masked. Overall, both sequence production and bioinformatics influence the distribution of samples, and both should be rigorously standardized if we are to expect distributions whereby samples from the same individual cluster irrespective of who runs the analysis. Reprocessing of partial-length metabarcoding partner 16S rRNA sequence files with a single bioinformatic pipeline highlighted the biases due to the use of different databases for taxonomical annotation. The results highlighted that these profiles dissimilarities between P1, P2, P4, and P5 are due to bioinformatic differences in the way taxonomical annotation is carried out, which explains why the use of a single bioinformatic procedure homogenizes the alpha- and beta-diversity outputs. It has been previously shown that in many instances Greengenes, SILVA, and RDP cannot be mapped reliably to one another^74^, thus explaining much of the dissimilarity observed before reprocessing of sequences using only the RDP database for taxonomic annotation. However, despite the use of a single bioinformatic pipeline and similar taxonomic annotation databases, none of the bacterial genus profiles allow an inter-individual variance to be reached that is lower than the inter-laboratories variances.

The use of full-length 16S rRNA gene in LUMI-Seq® allows minimization of the presence of exclusive bacterial genera and improvement of the identification of unclassified reads, which tends to provide profiles with higher similarity with the ones provided by MGP and P5 compared to other partial-length metabarcoding partners. As previously demonstrated, the average bacterial species alpha-diversity as measured by the Shannon index in full-length was higher than that in the partial-length metabarcoding partners^23^. However, the relative proportion of unclassified bacterial sequences was high, which may be due to the missing information in the full-length 16S rRNA gene database used. We also noticed the absence of the genus *Pseudomonas* in the mock samples as well as *Alistipes* in S1_1 and *Bifidobacterium* in all samples except for S1_1 and S5_2, while they were identified by all other partners. These latter results contradict what has been reported by Jeong et al. using a similar methodology applied to gut microbiota profiling. Here again, the primer choice may have had a strong impact.

## Conclusions

Previous inter-laboratories studies reported biases in bacterial taxonomic profiling, following the transfer of different raw biospecimens^77^, human stool samples^36^, to raw 16S rRNA sequences obtained from mock microbial community samples^78^. Here, technical replicates of DNA extracted from human stool samples were transferred as aliquots to different laboratories, for sequencing and analysis. This approach allowed us for the first time to characterize specific biases due to library preparation and sequence production all the way to the bioinformatics steps, without having to consider the sample collection or DNA extraction protocols. Reprocessing of raw 16S rRNA sequences using a single bioinformatic pipeline also allowed measurement of biases specifically due to the bioinformatics pipeline.

This multi-center evaluation study of gut microbiome profiling reveals major biases mainly due to library preparation and the databases used for taxonomic annotation in bioinformatic pipelines for partial-length metabarcoding. Whereas biases due to library preparation have been evaluated^8^, the impact of the choice of databases only for bacterial genus taxonomic annotation has not been investigated much to date. To our knowledge, studies reporting bioinformatic pipeline benchmarks for metabarcoding profiling of bacteria from mock microbial communities^79^ or human stool samples^14^ were performed using similar reference databases. Yet it is known that databases cannot be mapped reliably onto one another^74^, and differences between bacterial profiling using mock communities have been evaluated^76^. This study highlights major differences in bacterial genus identification and relative abundance assessment due to the bioinformatic pipeline used, primarily due to the choice of the database used for taxonomic annotation. According to the choices made concerning primer design, PCR amplification protocol in sequencing strategy as well as taxonomic reference database and sequence homology threshold used in bioinformatic pipeline, we recommend the systematic use of *in-silico* methodology allowing to test the relevance of these combinations of choice according to the objective of the study.

This study also reveals that the use of a single bioinformatics pipeline does not allow reduction of the proportion of partner-exclusive bacterial genera in order to allow for a lower inter-individual variance than inter-laboratories variances between metabarcoding and metagenomic partners. For laboratories to control for the presence of false-positive or negative bacterial genera and to accurately evaluate their pipeline or set-up standard next-generation sequencing protocols and bioinformatic pipeline, publicly or commercially available reference biospecimens, cells, and DNA reagents should be used. Such gut-representative DNA mock community standards have recently been developed for the microbiome field^80^ and others have been made commercially available by companies such as ATCC (MSA-1006). Other companies, such as Zymo Research, can also provide gut cell microbiome standard and fecal sample references (ZymoBIOMICS). As previously highlighted, the use of these standards is critical to build-up clinical microbiota profiling or use in research laboratories to improve publication reproducibility as well as transportability of methods and results to routine practice^81^. In the near future, raw metabarcoding or metagenomics DNA sequences obtained from mock microbial communities representative of stool specimens or fecal samples, made available in publications with the pipeline being reported as set by the STORMS initiative^82^, may also be systematically used by laboratories for pipeline evaluation. If microbiome profiling is ever to be made available on the dashboard of clinicians, standardizable and inter-laboratory homogeneity of outputs will be crucial features.

### Supplementary Information

Supplementary Information 1.

Supplementary Table 4.

Supplementary Table 5.

## Data Availability

The datasets supporting the conclusions of this article, Metagenomic and metabarcoding FastQ files, are available in NCBI BioProject under accession number PRJNA911046.

## Abbreviations

IGC2: Integrated gene catalogue 2
IHMS: International human microbiome standards
INRAE: Institut nationale de recherche pour l'agriculture, l'alimentation et l'environnement
MGP: Metagenomic profiling
MSP: Metagenomic species
PCoA: Principal coordinate analysis
