# Supplementary material for: Multicenter evaluation of gut microbiome profiling by next-generation sequencing reveals major biases in partial-length metabarcoding approach
Source: Sci Rep. 2023 Dec 18;13:22593. doi: 10.1038/s41598-023-46062-7 (PMC10730622; doi:10.1038/s41598-023-46062-7)
Supplement: Supplementary file 1 — Supplementary Information 1. [file 41598_2023_46062_MOESM1_ESM.pdf]

Supplementary Tables

<u>Supplementary Table 1</u>: Metabarcoding participant materials and methods details for microbiome profiling as reported by P1 to P6. QC: quantity control, PC: purity control, SC: size control; std: standard deviation, PE: pair-end, SE: single-end, bp: base pair, std: standard deviation, OTU: operational taxonomic unit, ASV: amplicon sequence variant, NA: non-applicable

Participant	P1	P2	P3	P4	P5	P6
DNA quality control	QC, PC, SC	QC, PC	QC	QC	QC	QC
**Amplified 16S rRNA**						
Platform (sequencer)	MiSeq (Illumina)	MiSeq (Illumina)	MiSeq (Illumina)	MiSeq (Illumina)	MiSeq (Illumina)	NextSeq500 (Illumina)
Hypervariable region	V3-V4	V3-V4	V3-V4	V3-V4	V3-V4	V1-V9
Input DNA amount (ng)	12	25	5	12.5	12.5	0.5
Addition of control samples	Yes	No	Yes	Yes	Yes	Yes
Primers used (annealing temperature)	MuyzerP/ 784R (65°C)	MuyzerP/ 784R (68°C)	343F/ V4R (50°C)	341F/806R (55°C)	341F/805R (55°C)	27F/1492R (55°C)
No. of PCR cycles	30	30	30	33	25	In-house design
DNA polymerase (proofreading)	Accustart II PCR toughmix (vwr)	MTP Taq DNA Polymerase (Sigma-Aldrich)	Pfu (Promega)	KAPA HiFi HotStart ReadyMix (Roche)	KAPA HiFi HotStart Ready Mix (Roche)	In-house design
Control of library quantity/size	QC, SC	QC, PC, SC	QC, SC	QC, SC	QC, SC	QC, SC
Sequencing mode PE/SE (read size in bp)	PE (2x300)	PE (2x300)	PE (2x250)	PE (2x300)	PE (2x250)	PE (2x150)
Kit used for libraries construction	No kit	No kit	Proprietary design	Illumina sequencing method	Nextera XT Index kit (Illumina)	Proprietary design
**Bioinformatic analysis**						
Demultiplexing software	Bcl2fastq (v2.20.0)	Splitbc.pl: home-made Perl script shared with FROGS	CASAVA (v1.0)	bcl2fastq	bcl2fastq	bcl2fastq
Mean raw-reads number per sample (std.)	149,627 (21,571)	92,149 (20,616)	56,595 (5,180)	249,676 (63,053)	287,684 (29,216)	3,935,462 (5,416,829)
Sequence quality control software	Fastqc (v0.11.2)	FastQC (v11.7) MultiQC (v1.5)	FastQC (v0.11.5)	FastQC (v0.11.5) Fastp (v0.12.6)	FastQC (v0.11.8)	FastQC (v0.11.5) Fastp (v0.12.6)
Read-merging software – overlap (bp)	Mothur (v1.39.5)	Vsearch (v2.6.2) – 30	FLASH (v1.2.11) - 30	PEAR (v0.9.10) – 7	Mothur (v1.40.0)	SPAdes (v 3.14.1)
Sequence removal	Homopolymers (>8 pb) + chimeric	Read size + chimeric + rare OTUs (present in at least 3 samples)	Read quality (<Q30) + chimeric	rare OTUs (<0.005%)	Chimeric + rare OTUs (cluster with one sequence)	Short/Not full-length 16S sequences
Sequence-denoising software	Mothur (v1.39.5)	preprocess.py	NA	NA	Mothur (v1.40.0)	V-Xtractor (v2.1)
Mean read length after quality control (pb)	141.6	410	408	450	448	~ 1,500 bp
Mean reads number per sample after quality control	112,765	52,283	55,775	246,854	204,916	2,300,000
Data analysis software	Mothur (v1.39.5)	FROGS (v3.1)	QIIME (v1.9.1)	QIIME (v1.9.1)	Mothur (v1.40.0)	DADA2 (v1.18.0)

Clustering strategy (OTU/ASV)	OptiClust (Mothur) (OTU)	Swarm (v2.2.2) (OTU)	Uclust (v1.2.22q) (OTU)	Usearch (v6.1) (OTU)	Opticlust (Mothur) (OTU)	DADA2 (ASV)
16S rRNA taxonomic classifier	Mothur (v1.39.5)	NCBI blastn+ 2.7.1	RDP_classifier (v2.2)	RDP_classifier (v2.2)	Greengenes (v13.5.99)	Naive Bayesian classifier
16S rRNA reference database	Greengenes (v.13.8.99)	SILVA (v132) filtered on pintail greater than 50	Greengenes (v13.8)	SILVA (v132)	RDP database (v16)	Proprietary 16S database

Supplementary Table 2: Sequence of the forward and reverse primers used to amplify V3-V4 (P1-P5) and V1-V9 (P6) hypervariable regions of the 16S rRNA gene

Metabarcoding partners	Forward primer	Reverse primer
**P1**	ACGGRAGGCAGCAG	TACCAGGGTATCTAATCCT
**P2**	ACGGRAGGCAGCAG	TACCAGGGTATCTAATCCT
**P3**	TACGGRAGGCAGCAG	CTACCNGGGTATCTAAT
**P4**	CCTACGGGAGGCAGCAG	GGACTACHVGGGTWTCTAAT
**P5**	CCTACGGGNGGCWGCAG	GACTACHVGGGTATCTAATCC
**P6**	AGAGTTTGATCCTGGCTCAG	GGTTACCTTGTTACGACTT

<u>Supplementary Table 3</u>: Main bioinformatic pipeline steps followed when reprocessing short-read metabarcoding partner datasets

Bioinformatic analysis	P1	P2	P3	P4	P5
Mean raw-reads number per sample (std.) per partner	149,627 (21,571)	92,149 (20,616)	56,595 (5,180)	249,676 (63,053)	287,684 (29,216)
Sequence quality control software	FastQC v0.11.9				
Read-merging software – overlap (bp)	PEAR 0.9.11				
Sequence removal	Read quality (< Q30) + read length (> 250) + chimeric				
Sequence denoising software	vsearch v2.21.1				
Mean read length after quality control (pb)	250				
Mean reads number per sample after quality control per sample (std.) per partner	62,433	40,588	29,927	35,286	199,102
Data analysis software	vsearch v2.21.1				
Clustering strategy (OTU/ASV)	vsearch v2.21.1 – unoise3 (ASV)				
16S rRNA taxonomic classifier	RDP Classifier 2.13				
16S rRNA reference database	RDP Classifier 2.13				

Supplementary Figures

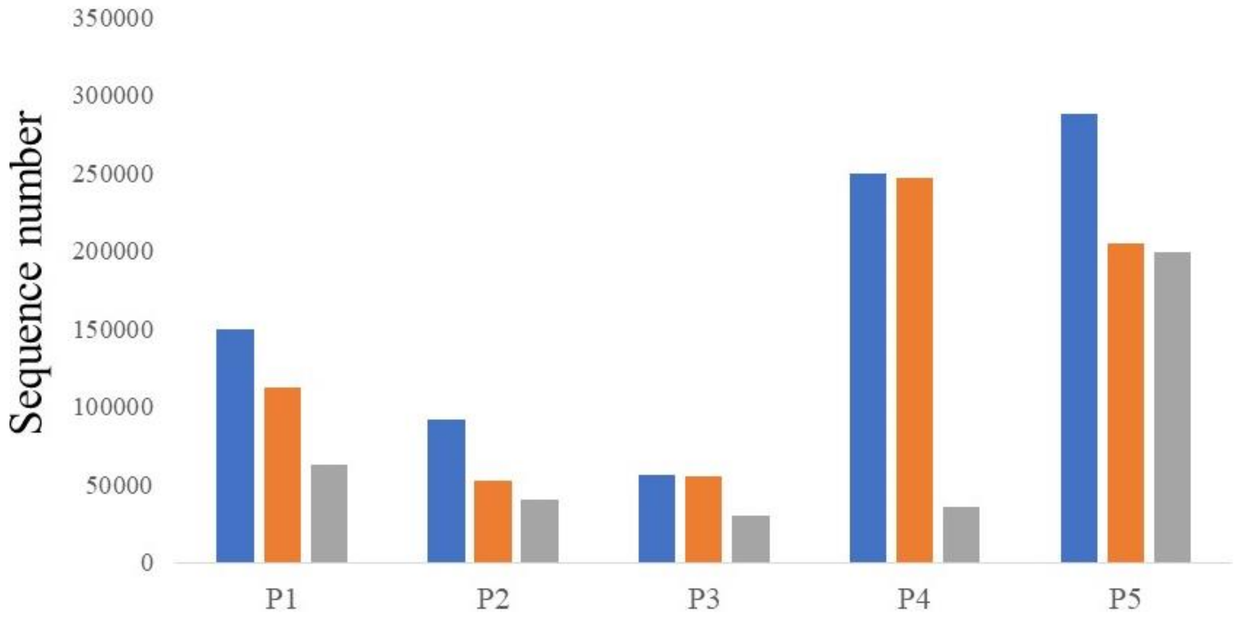

<u>Supplementary Figure 1:</u> Bar chart representing number of raw (blue) and trimmed (orange) reads obtained by partial-length metabarcoding partners (P1 to P5) and following use of a single and unique bioinformatic pipeline (grey).

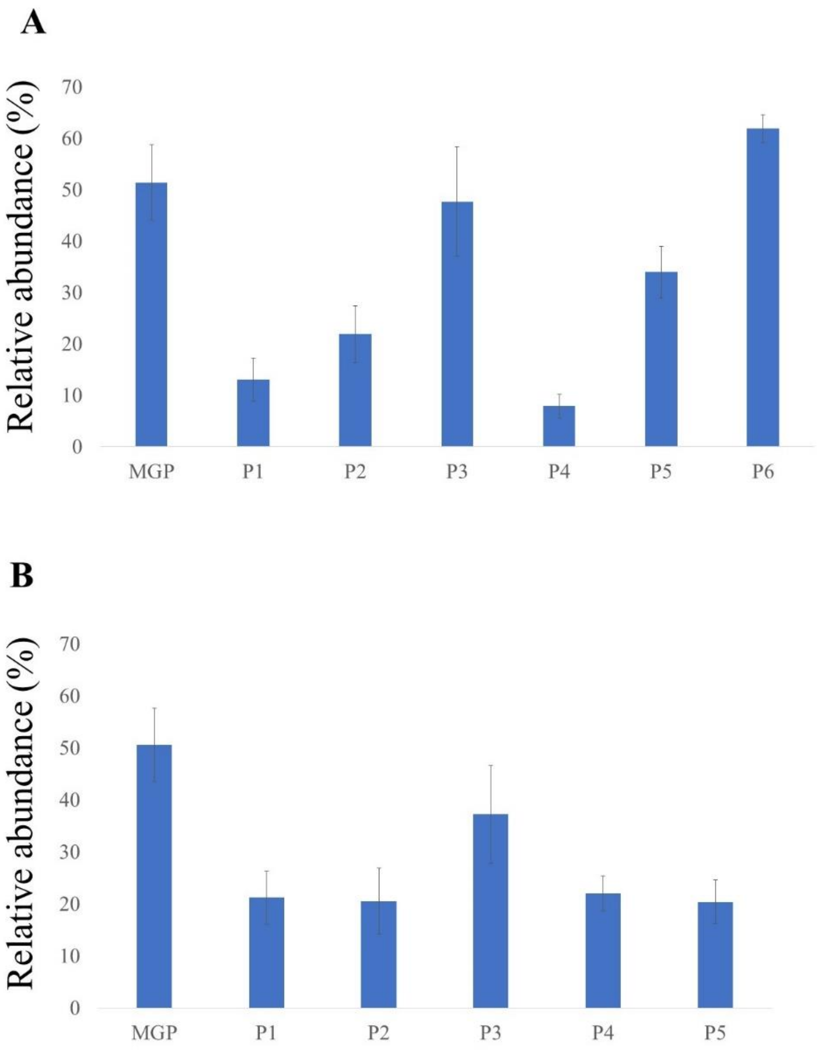

Supplementary Figure 2: Bar chart representing relative abundances (%) and standard deviation of unclassified bacterial genera identified in all partners considering fecal samples, with the original partner dataset (**A**) and after data reanalysis of short-read metabarcoding partner (**B**).

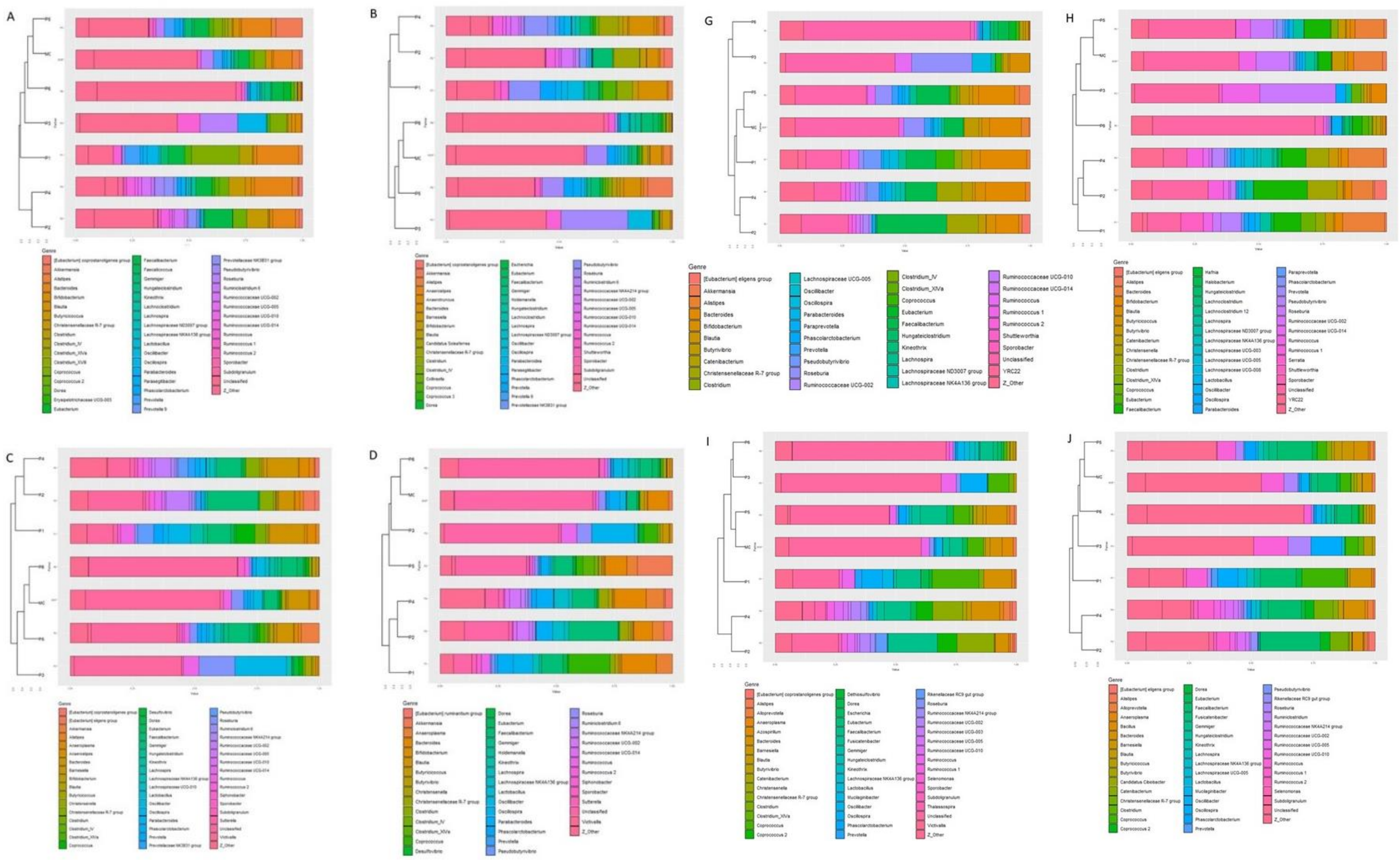

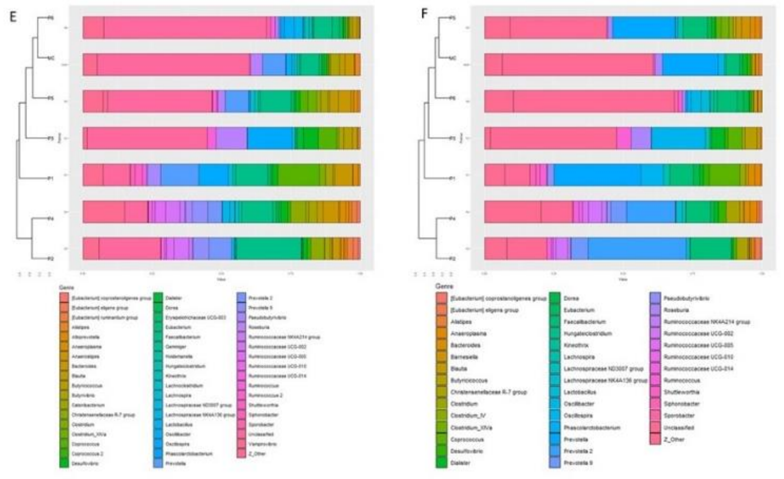

<u>Supplementary Figure 3</u>: **S**tacked bar plot with hierarchical clustering (dendrogram), based on calculation of the Bray–Curtis dissimilarity index calculated between feacal samples. Average relative abundance of microbial genera in the fecal samples among triplicate samples for S1_1 (**A**), S1_2 (**B**), S2_1 (**C**), S2_2 (**D**), S3_1 (**E**), S3_2 (**F**), S4_1 (**G**), S4_2 (**H**), S5_1 (**I**), and S5_2 (**J**) obtained by shotgun (MGP) and 16S rRNA (P1 to P6) sequencing strategy.

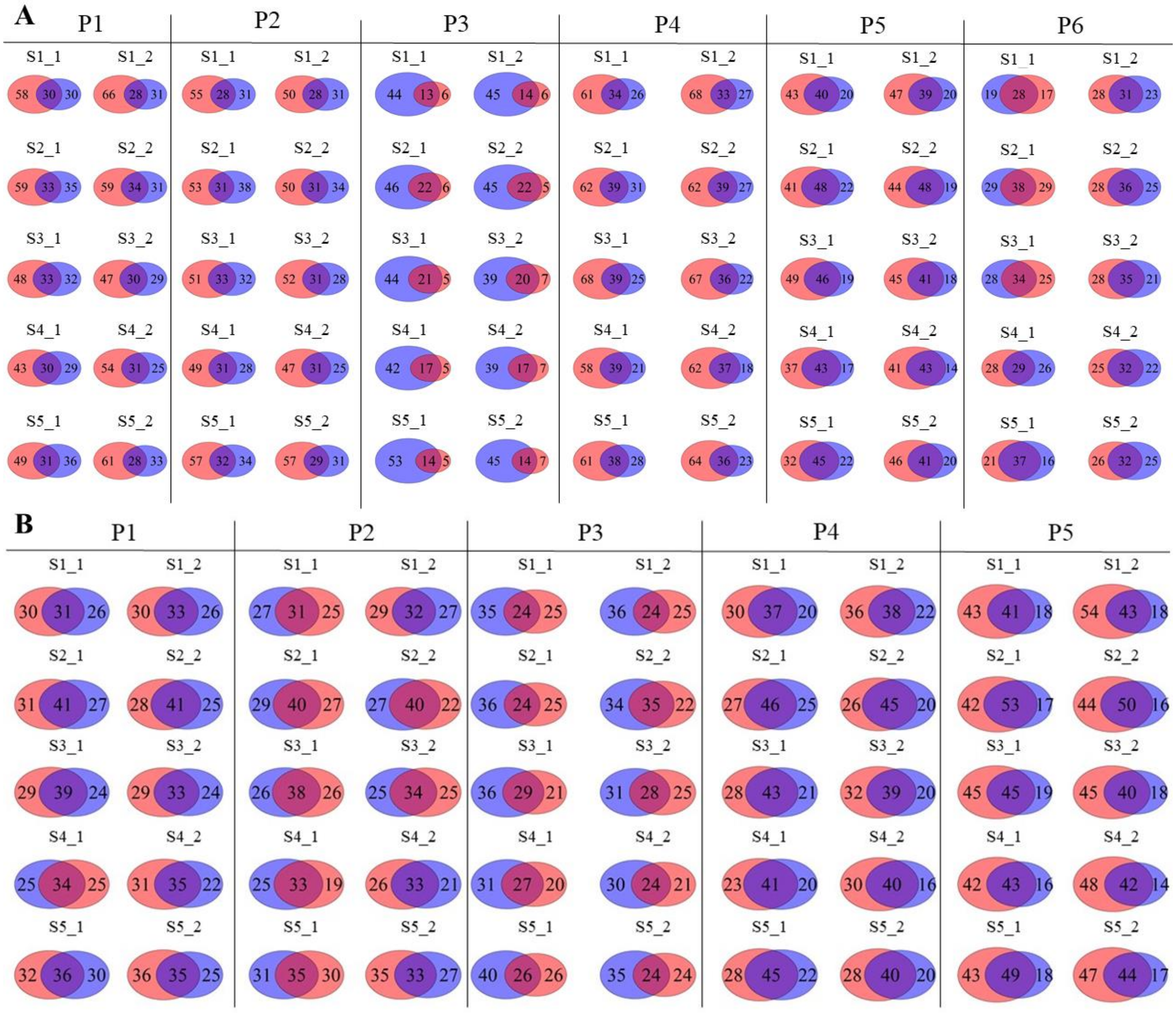

Supplementary Figure 4: VennDiagram of number of bacterial genera exclusively identified in 16S rRNA sequencing partners, in MGP only, or shared between both for all samples and partners, with partner (**A**) and similar (**B**) bioinformatic pipeline. Red circle: present in 16S rRNA sequencing partner only, blue circle: present in MGP only, purple/pink circle: present in both datasets.

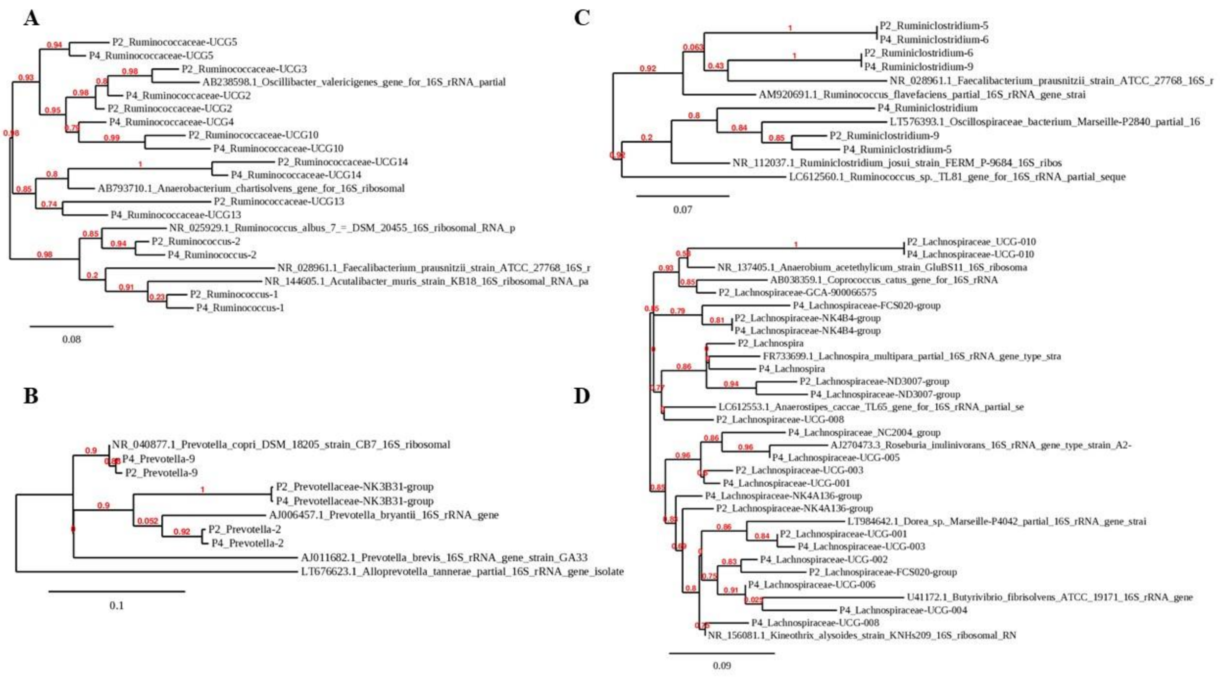

<u>Supplementary Figure 5</u>: Phylogenetic trees built from P2 and P4 partner-exclusive identified bacterial genera considering the 16S rRNA sequences from (**A**) *Ruminococcus*, (**B**) *Prevotella*, (**C**) *Ruminiclostridium*, and (**D**) *Lachnospira* provided by partner and reference 16S rRNA sequences from same genus download from the NCBI.

A

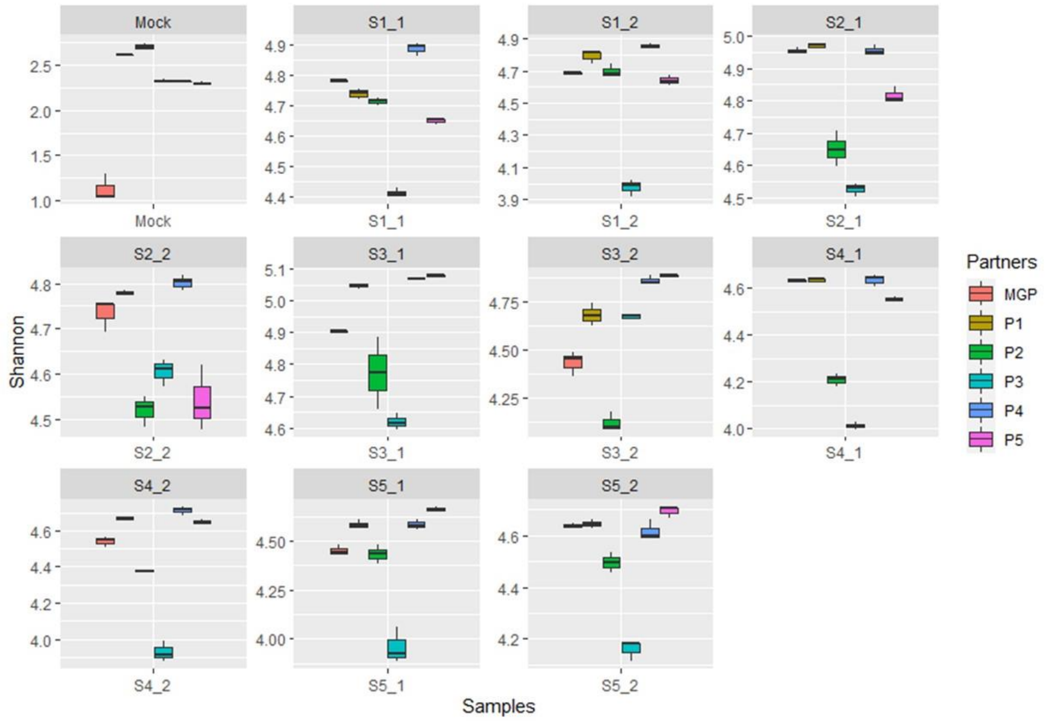

B

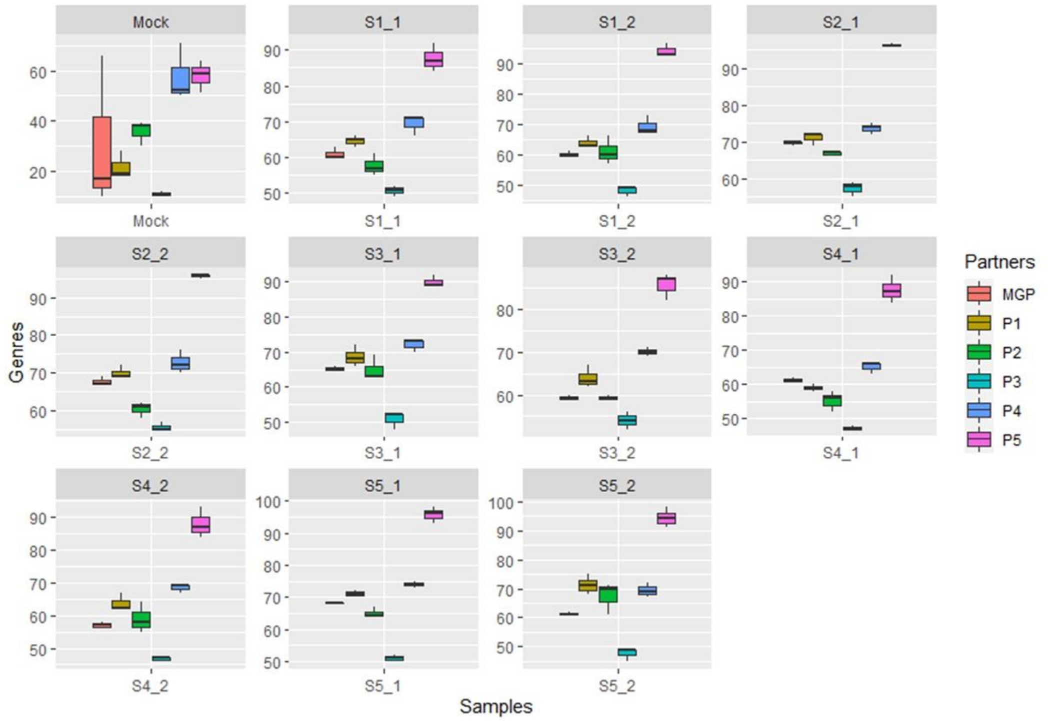

<u>Supplementary Figure 6</u>: Boxplots of the alpha-diversity comparative analysis base on microbial genus number identified in samples for all partners (MGP, P1 to P6) following reanalysis of the

raw dataset with a single bioinformatic pipeline**.** (**A**) Average Shannon index calculated by partner on the basis of bacterial species diversity. (**B**) Average number of bacterial genus number identify per triplicate sample for all partners (MGP, P1 to P5) with the standard deviation.

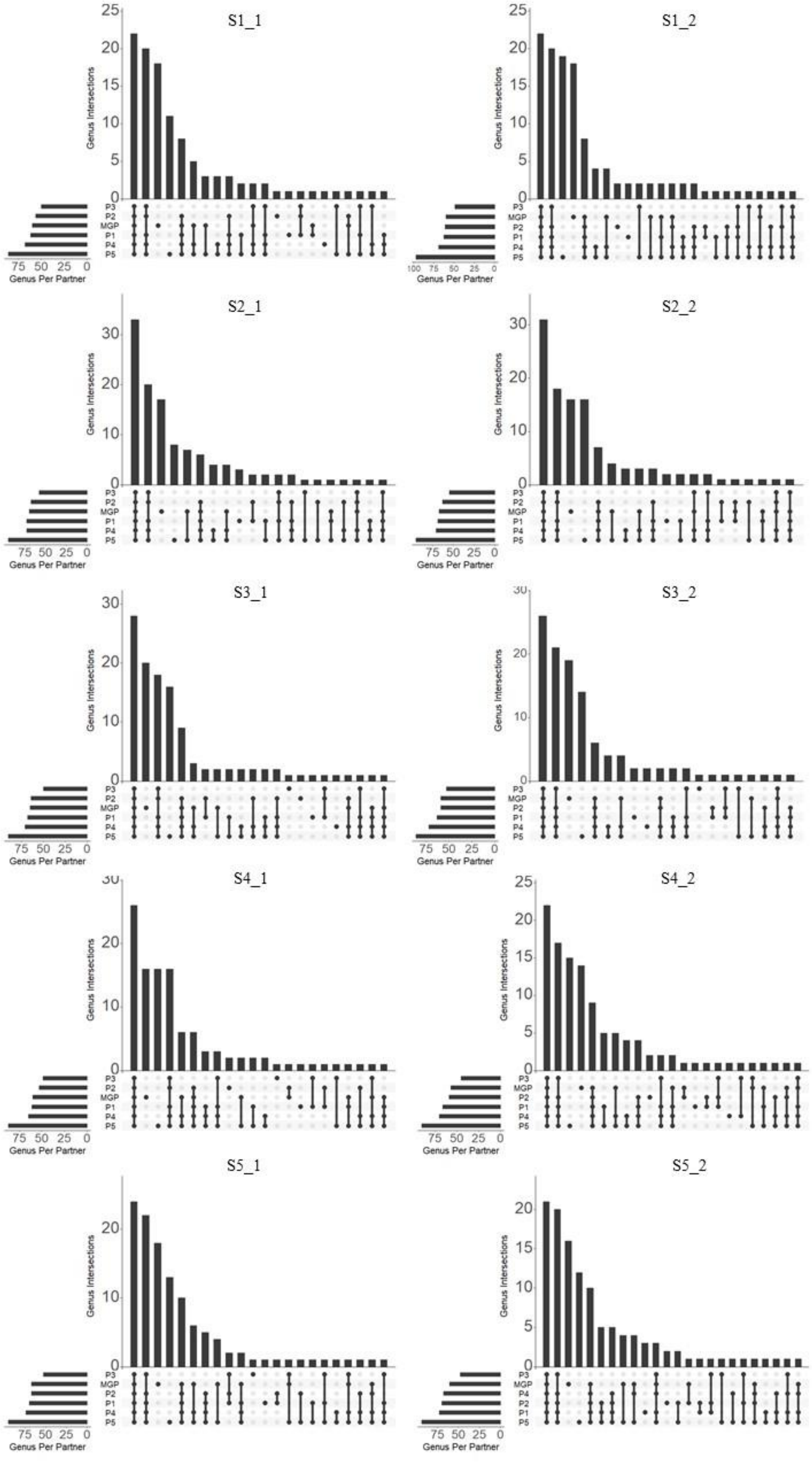

Supplementary Figure 7: : UpSet plot representing the number of bacterial genera in the human fecal samples (S1_1 to S5_2) exclusively identified by one partner or shared between multiple

partners (MGP, P1, P2, P3, P4, and P5) following data reanalysis of partial-length metabarcoding partners.
